# FSH-secreting Giant Pituitary Macroadenoma With Aggressive Clinical Behavior

**DOI:** 10.1210/jcemcr/luaf202

**Published:** 2025-09-24

**Authors:** Melina I Manolas, Zoe M Videlefsky, Janaki M Samavedam, Fnu Sapna, Beatrice Y Wong, Simona Stefan

**Affiliations:** Department of Internal Medicine, Division of Endocrinology, Diabetes, & Metabolism, Weill Cornell Medical College, New York, NY 10021, USA; Albert Einstein College of Medicine, Bronx, NY 10461, USA; Department of Internal Medicine, Montefiore-Einstein Medical Center, Bronx, NY 10461, USA; Department of Pathology, Montefiore-Einstein Medical Center, Bronx, NY 10461, USA; Department of Internal Medicine, Division of Endocrinology, Montefiore-Einstein Medical Center, Bronx, NY 10461, USA; Department of Internal Medicine, Division of Endocrinology, Montefiore-Einstein Medical Center, Bronx, NY 10461, USA

**Keywords:** functional gonadotroph adenoma, aggressive pituitary tumors, pituitary adenoma

## Abstract

We describe a case of a 51-year-old woman with a 15-year history of a functional gonadotroph adenoma (FGA) secreting FSH, characterized by persistent hormonal hypersecretion, progressive tumor growth despite multiple surgeries, and anterior hypopituitarism. She initially presented at age 35 with menorrhagia and diplopia and underwent transsphenoidal surgery (TSS) abroad. Upon relocating to the United States 3 years later, she presented with recurrent menorrhagia, elevated FSH and estradiol, and a 3.2 cm invasive macroadenoma, requiring repeat TSS. Pathology demonstrated isolated FSH immunopositivity with a low Ki-67 index (≤3%). The postoperative course was complicated by central hypothyroidism and adrenal insufficiency. Despite a trial of bromocriptine, she experienced ongoing menorrhagia and tumor growth to 6.8 cm, requiring a third TSS and hysterectomy. Management was further complicated by inconsistent follow-up and hesitancy toward recommended radiotherapy. Fifteen years after her initial presentation, the residual tumor exceeded 8 cm, causing seizures, hallucinations, and vision loss despite persistently low Ki-67 proliferation (<3%). This case highlights the diagnostic and therapeutic challenges of FGAs, especially when tumor behavior is discordant with low-grade histologic features. It underscores the need for improved prognostic markers and earlier consideration of multimodal therapy, including radiotherapy and chemotherapy, in refractory cases.

## Introduction

Gonadotroph pituitary adenomas are common but are typically nonfunctioning, often diagnosed incidentally or due to symptoms from mass effect. In contrast, functional gonadotroph adenomas (FGAs) are rare, accounting for less than 1% of all pituitary adenomas [1]. FGAs secrete biologically active gonadotropins, usually FSH, and often present as macroadenomas, leading to symptoms of both mass effect and hormonal excess. Typical biochemical findings include elevated FSH, low LH, and increased estradiol in females. In males, testosterone levels may range from low to normal or elevated [1]. Clinical manifestations vary depending on sex and menopausal status. Premenopausal women may experience ovarian hyperstimulation syndrome and menstrual irregularities, while men may present with macroorchidism and increased spermatogenesis [1, 2]. In postmenopausal women, the diagnosis is often delayed or overlooked due to physiologically elevated gonadotropin levels, which can mask biochemical abnormalities [3]. Surgery remains the standard treatment, while medical therapies such as dopamine agonists or GnRH agonists have demonstrated inconsistent results [1].

Here we present the 15-year clinical course of a woman with a functional FSH-producing macroadenoma characterized by progressive, recurrent tumor growth despite multiple surgical resections and low mitotic index.

## Case Presentation

A 35-year-old woman initially presented with diplopia and heavy menstrual bleeding in her home country of Jamaica. Imaging reportedly revealed a pituitary macroadenoma, and she underwent her first transsphenoidal surgery (TSS) in Jamaica at age 35, with subsequent improvement in symptoms. She has no history of fertility issues and had 3 children via spontaneous vaginal delivery. She has no family history of pituitary tumors or endocrine disorders.

**Figure 1.**
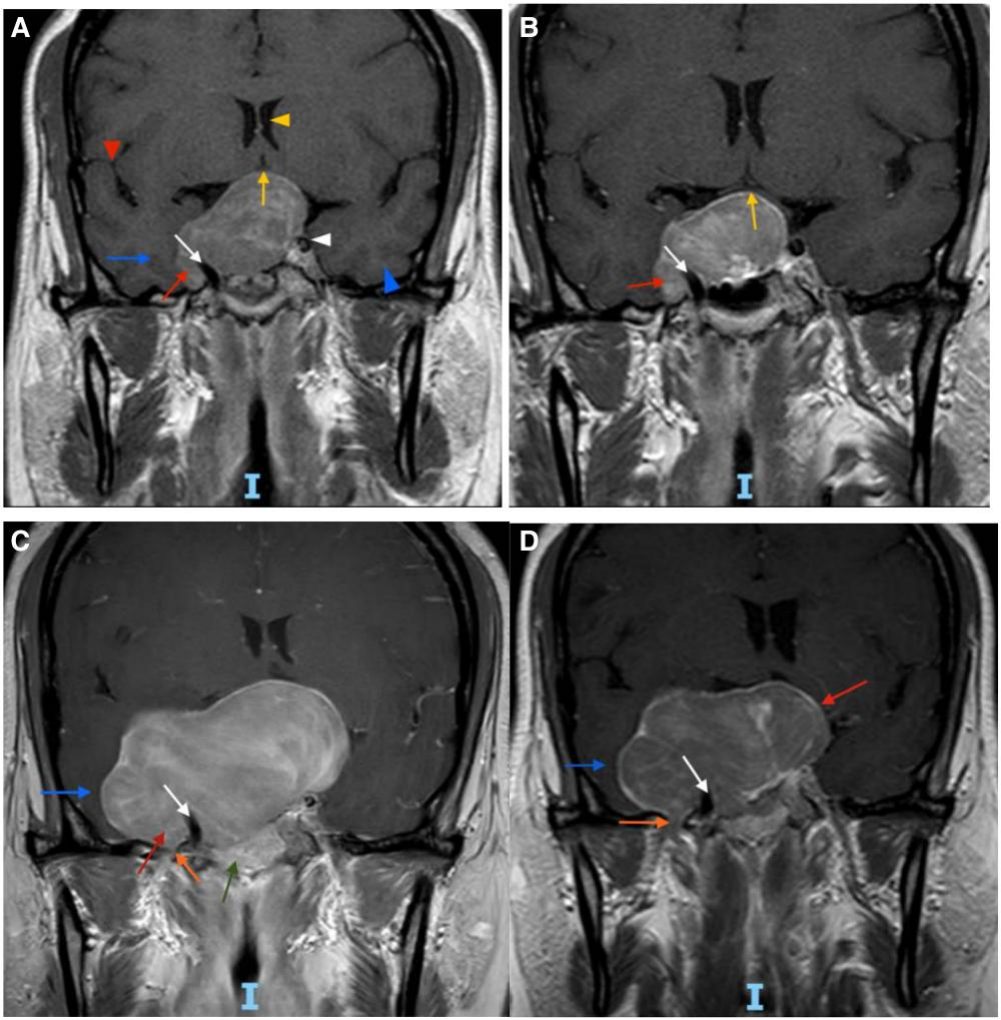
Serial magnetic resonance imaging brain images showing change in tumor size before and after second and third TSS. (A) Age 40, pre-second TSS: T1-weighted postcontrast image demonstrates persistent pituitary macroadenoma following initial TSS at age 35. Tumor is elevating the optic chiasm (yellow arrow), encasing the cavernous segment of the right internal carotid artery (white arrow) and invading into the right cavernous sinus (red arrow). There is displacement of the right temporal lobe (blue arrow). Normal appearing internal carotid artery, cavernous sinus, and temporal lobe on the left side (white and blue arrowheads). Lateral ventricles (yellow arrowhead) appear symmetric as do bilateral sylvian fissures (red arrowhead). (B) Age 40 post-second TSS: T1 postcontrast image several months after second TSS. Tumor is smaller in size with decreased mass effect on the optic chiasm (yellow arrow). The mass continues to invade the right cavernous sinus (red arrow) and encase the right internal carotid artery (white arrow). (C) Age 47, pre-third TSS: T1-weighted postcontrast image shows interval growth of the mass 6 years after second TSS. Tumor is invading the right cavernous sinus to a greater degree (red arrow) and causing more significant mass effect on the right temporal lobe (blue arrow). There is also extension inferiorly into the right sphenoidal sinus (green arrow) and involvement of the foramen ovale (orange arrow). (D) Age 47, post-third TSS: T1-weighted postcontrast image several months after third TSS. Notably, there is decompression of the left portion of the mass (red arrow). However, there is persistent invasion into the right cavernous sinus and encasement of the right carotid artery (white arrow), extension through the foramen ovale (orange arrow), and mass effect on the right temporal lobe (blue arrow). Abbreviation: TSS, transsphenoidal surgery.

At age 40, 3 years after immigrating to the United States, she developed recurrent menorrhagia. Magnetic resonance imaging (MRI) of the pituitary revealed a 3.2 cm invasive macroadenoma (Fig. 1, panel A), prompting a second TSS. Her postoperative clinical course was complicated by central hypothyroidism and adrenal insufficiency. Follow-up imaging showed decreased tumor size and reduced mass effect on the optic chiasm (Fig. 1, panel B).

**Figure 2.**
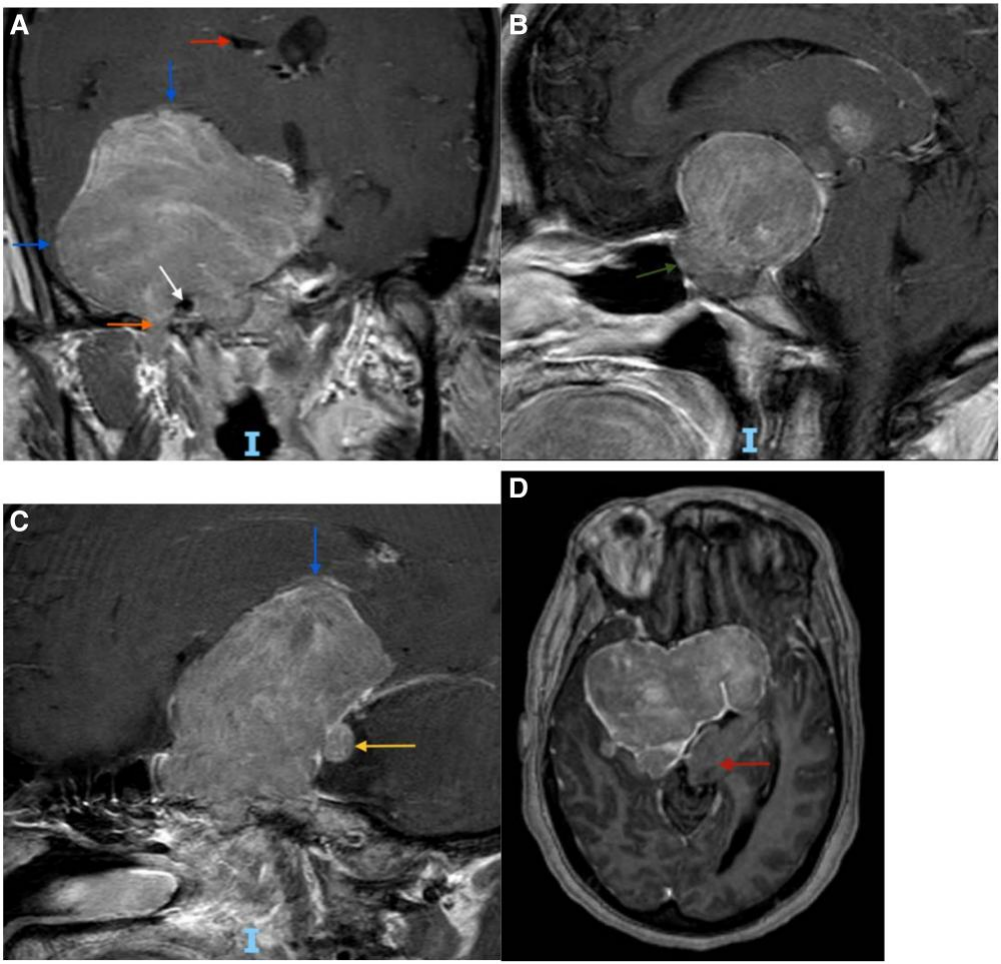
Magnetic resonance imaging brain (coronal, sagittal, and axial views) at age 51, taken during the patient's most recent clinical presentation, demonstrates tumor progression since the third TSS at age 47. (A) T1-weighted postcontrast image demonstrates interval growth of the tumor with significant mass effect on the right temporal lobe and the right inferior frontal lobe (blue arrows). There is posterior extension through the foramen ovale (orange arrow) and invasion of the right cavernous sinus with encasement of the right internal carotid artery (white arrow). There is also partial effacement of the right lateral ventricle (red arrow). (B) T1 postcontrast sagittal view image shows tumor extension into the sphenoidal sinus (green arrow). (C) T1 postcontrast saggital view image reveals displacement of the inferior frontal lobe (blue arrow) and invasion into the posterior fossa (yellow arrow). (D) Axial image shows leftward displacement of the brainstem (red arrow). Abbreviation: TSS, transsphenoidal surgery.

She experienced persistent menorrhagia, with bleeding nearly 20 days per month and frequent hospitalizations for symptomatic anemia. At age 46, she underwent a total abdominal hysterectomy with bilateral salpingo-oophorectomy (TAH-BSO). One year later, at age 47, surveillance MRI demonstrated residual tumor growth to 6.8 cm with new extension into the sphenoid sinus and foramen ovale (Fig. 1, panel C), prompting a third TSS. Despite treatment, and complicated by gaps in medical care, her tumor progressed to >8 cm with extension through the foramen ovale, posterior fossa invasion, and displacement of the brainstem (Fig. 2) with worsening vision loss, seizures, and auditory hallucinations by age 51.

## Diagnostic Assessment

Due to lack of biochemical, imaging, or pathology records from her initial TSS performed abroad, the available diagnostic evaluation began after the patient established care in the United States Initial hormonal assessment in our medical system following her first TSS revealed a significantly elevated FSH level of 74.5 mIU/mL (International System of Units [SI]: 74.5 IU/L) (reference range [RR]: follicular, days 2-3: 3.0-14.4 mIU/mL [SI: 3.0-14.4 IU/L]; midcycle: 5.8-21.0 mIU/mL [SI: 5.8-21.0 IU/L]; luteal: 1.2-9.0 mIU/mL [SI: 1.2-9.0 IU/L]), with cycle timing unclear. LH was 2.68 mIU/mL (SI: 2.68 IU/L) (RR: follicular, days 2-3: 1.1-11.6; midcycle: 17.0-77.0; luteal: 1.0-14.7), and estradiol was elevated at 1800 pg/mL (SI: 6607 pmol/L) (RR: 6.0-490 pg/mL [SI: 22-1798.8 pmol/L]), concerning for a functional, FSH-producing adenoma (Table 1). α subunit was 1.0 ng/mL (SI: 1.0 μg/L) (RR: ≤1.5 ng/mL [SI: ≤1.5 μg/L] for premenopausal women). Additional laboratory tests confirmed central hypothyroidism, central adrenal insufficiency, and low IGF-1 and prolactin levels.

**Table 1.** Biochemical evaluation over time

Hormone tested	Normal range	Initial presentation in U.S. (after first TSS)	After second TSS	Before third TSS (and after BSO)	After third TSS	Most recent admission
FSH	Premenopausal: <20 mIU/mL (<20 IU/L) Postmenopausal: <150 mIU/mL (<150 IU/L)	74.5 mIU/mL (74.5 IU/L)	36.4 mIU/mL (36.4 IU/L)	>150.0 mIU/mL (>150.0 IU/L)	>150.0 mIU/mL (>150.0 IU/L)	ND
LH	Premenopausal: <10 mIU/mL (<10 IU/L) Postmenopausal: <60 mIU/mL (<60 IU/L)	3.25 mIU/mL (3.25 IU/L)	1.64 mIU/mL (1.64 IU/L)	3.4 mIU/mL (3.4 IU/L)	4 mIU/mL (4 IU/L)	ND
Estradiol	6.0-490 pg/mL (22.0-1798.8 pmol/L)	1800 pg/mL (6607.8 pmol/L)	320 pg/mL (1174.7 pmol/L)	<25 pg/mL (91.8 pmol/L)	ND	ND

Values in parentheses are International System of Units.

Abbreviations: BSO, bilateral salpingo-oophorectomy; ND, no data; TSS, transsphenoidal surgery.

**Figure 3.**
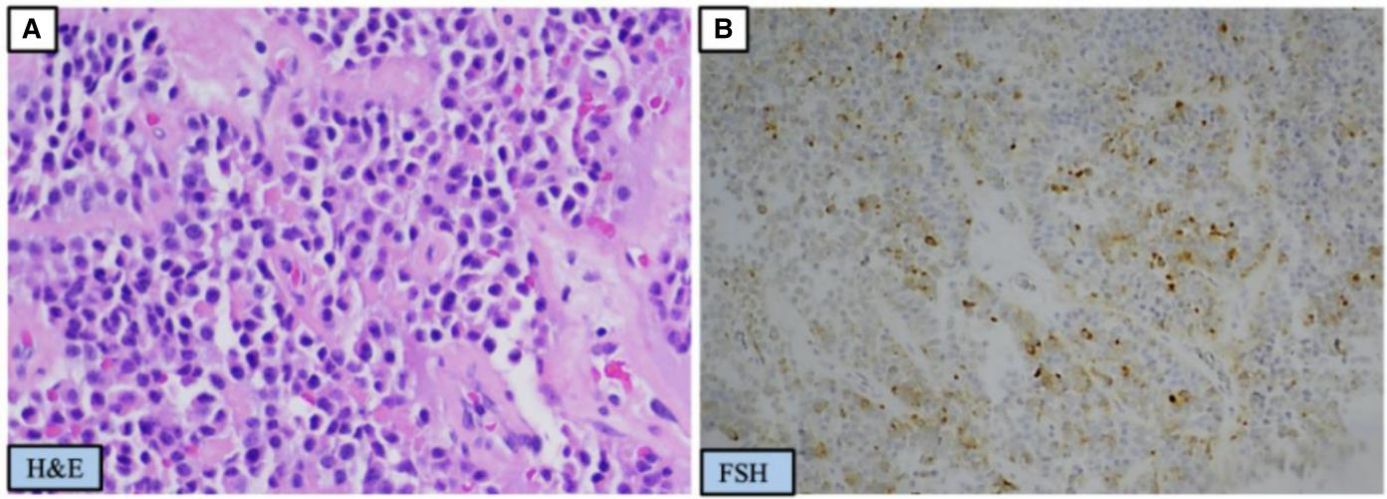
Surgical pathology from the patient's second transsphenoidal surgery. (A) Hematoxylin and eosin staining of neoplastic pituitary tissue forming solid nests. (B) Positivity of neoplastic cells with FSH.

Repeat MRI confirmed a 3.2 cm pituitary macroadenoma, leading to her second TSS at the age of 40. Pathology demonstrated isolated FSH immunopositivity (Fig. 3), a Ki-67 proliferation index of approximately 3%, and rare p53-positive nuclei. Transcription factor analysis was not performed. FSH and estradiol levels had decreased to 36.4 mIU/mL (SI: 36.4 IU/L) and 320 pg/mL (SI: 1174.7 pmol/L), respectively.

Given ongoing symptoms of menorrhagia, pelvic imaging was obtained. Pelvic ultrasound showed a fibroid uterus, heterogeneous and vascular thickened endometrium, and bilaterally enlarged ovaries (right: 27.5 cm^3^; left: 19.5 cm^3^), exceeding the typical premenopausal ovarian volume. Doppler flow was normal, and no multicystic features were noted. Endometrial biopsy revealed proliferative endometrium with tubal metaplasia. A trial of bromocriptine 2.5 mg daily for nearly 7 years did not provide any clinical benefit. She later declined oral progesterone but received a single dose of intramuscular medroxyprogesterone acetate, which provided temporary symptomatic relief. Ultimately, at age 46, she underwent TAH-BSO. Surgical pathology confirmed uterine fibroids and benign overgrowth of the endometrium. Additionally, ovarian pathology revealed a serous cystadenofibroma on the right, a vascular hamartoma on the left, as well as follicle cysts and mild congestion. Postoperative labs showed menopausal-range FSH levels >150 mIU/mL (SI: >150 IU/L) and estradiol levels of <25 pg/mL (SI: <91.8 pmol/L).

Subsequent pituitary imaging 1 year later revealed progressive tumor enlargement to 6.8 cm (Fig. 2, panel C), necessitating a third TSS at the age of 47. Pathology showed dual LH and FSH expression, with a Ki-67 proliferation index of less than 1%. By the age of 51, the patient's tumor exceeded 8 cm, encasing the right internal carotid artery and invading the skull base (Fig. 2).

## Treatment

Over a 15-year period, the patient underwent 3 transsphenoidal surgeries (at ages 35, 40, and 47). Hormonal replacement included levothyroxine 100 mcg daily and prednisone 5 mg daily. She repeatedly declined medical recommendations for adjuvant radiotherapy. Her clinical course was further complicated by social barriers to care, including limited assistance navigating the healthcare system, resulting in prolonged gaps in medical care.

## Outcome and Follow-up

The patient has experienced progressive neurocognitive decline and relies on surrogate decision-making. Multidisciplinary follow-up continues to discuss further surgery, radiation therapy, and/or chemotherapy with temozolomide.

## Discussion

FGAs are rare, hormonally active pituitary tumors that typically secrete FSH and can present with diverse clinical manifestations depending on sex and hormonal context. Premenopausal women often present with menstrual irregularities, menorrhagia, or ovarian hyperstimulation syndrome (OHSS) [1, 2].

Although OHSS is most commonly associated with assisted reproductive technologies, it may occasionally occur spontaneously in reproductive-aged women with FGAs and serve as an initial indicator of an underlying pituitary tumor. In OHSS, excess secretion of FSH leads to enlarged multicystic ovaries, often exceeding 10 cm in diameter; elevated estradiol levels; and symptoms such as menstrual irregularities, abdominal or pelvic pain, distension, and nausea [4]. Imaging typically shows bilaterally enlarged ovaries with thin-walled cysts lacking solid components. Histopathology reveals preserved ovarian architecture, multiple follicular cysts lined by granulosa cells, and absence of stromal hyperthecosis or neoplastic changes [4, 5]. In our patient, pelvic ultrasound demonstrated bilaterally enlarged ovaries but lacked multicystic features. However, surgical pathology identified multiple follicular cysts, consistent with hormonally driven ovarian hyperstimulation. These nuances underscore the variable phenotypic expressions of FGAs and the critical importance of early recognition to prevent potentially unnecessary gynecologic interventions. Although this patient ultimately underwent oophorectomy due to persistent menorrhagia, the absence of typical OHSS imaging findings highlights the diagnostic complexity associated with these tumors.

Notably, the tumor grew markedly, from 2.9 cm post-second TSS to 6.8 cm 1 year after oophorectomy, possibly reflecting loss of estradiol feedback and resultant unregulated pituitary FSH secretion. This pattern of tumor enlargement following removal of negative feedback after oophorectomy may be analogous to Nelson syndrome, in which corticotroph tumors enlarge after bilateral adrenalectomy due to loss of cortisol feedback. However, since the exact tumor size immediately prior to TAH-BSO is unknown, it is difficult to attribute the growth solely to this mechanism.

Surgical resection remains the standard of care for FGAs and often leads to hormonal normalization and symptom resolution. Although FGAs are typically slow-growing and responsive to surgery, a subset can behave aggressively, with rapid recurrence and local invasion. Wang et al reviewed several cases of FGAs in premenopausal women, observing that although most responded adequately to surgical resection, a subset required repeated surgeries, radiation, or additional medical therapy [2]. In this patient, serial imaging spanning more than a decade (Figs. 1 and 2) demonstrated progressive tumor growth to over 8 cm despite 3 surgical resections, with extensive local invasion and neurocognitive decline. Troullias et al's 2013 clinicopathological classification emphasizes the prognostic importance of tumor invasion, Ki-67 proliferation, and p53 expression in stratifying tumor behavior [6]. However, our patient's tumor demonstrated extensive growth and invasion despite a persistently low Ki-67 index (≤3%) and only focal p53 positivity. Such discordance between histological grading and clinical aggressiveness highlights the limitations in current prognostic markers, reinforcing the necessity of integrating clinical and imaging features in patient assessment. Similar discordance has been reported in other aggressive pituitary tumors with low Ki-67 and variable p53 expression [7, 8].

In refractory cases like this one, consideration of multimodal therapy, including radiotherapy and temozolomide, would be consistent with current recommendations for managing aggressive pituitary tumors [7]. The role of radiotherapy in FGA management remains unclear, though it has been employed in cases of tumor regrowth. While potentially beneficial, radiotherapy was declined by the patient in this case. Medical therapies—including dopamine agonists, somatostatin analogs, GnRH agonists, and antagonists—have shown variable efficacy. Dopamine agonists like bromocriptine can reduce FSH and estradiol levels in select cases [9], but they have not shown consistent benefit in reducing tumor size and did not alter the disease trajectory in our patient. Emerging therapies such as antivascular endothelial growth factor agents, immunotherapy, and tyrosine kinase inhibitors remain experimental in this context.

This case was limited by the absence of initial medical records from Jamaica, including surgical pathology and imaging, making it difficult to distinguish tumor recurrence from residual disease. However, the patient's clinical course in the United States following both a second and third TSS suggests aggressive residual disease. Tumor classification was further limited by the lack of transcription factor analysis. Additionally, significant social and systemic barriers, including limited healthcare navigation support and prolonged gaps in follow-up, contributed to delayed treatment and worsening neurologic outcomes.

This case underscores that aggressive clinical behavior in FGAs may not correlate solely with proliferative markers such as the Ki-67 index. It highlights the critical importance of prompt diagnosis, coordinated multidisciplinary care, and continuous, long-term follow-up, especially in patients with incomplete surgical resection of delayed diagnosis to improve patient outcomes.

## Learning Points

- FGAs typically secrete FSH, causing variable clinical presentations. Premenopausal women may experience menstrual irregularities or ovarian hyperstimulation, while postmenopausal women may have subtle or absent symptoms, complicating diagnosis.
- FGAs can exhibit rapid growth and recurrence despite a low mitotic index.
- Early multimodal intervention, including radiotherapy or chemotherapy, may be warranted in aggressive or recurrent FGAs.

## Contributors

All authors made individual contributions to authorship. M.I.M. and Z.M.V. developed the first draft of this manuscript, and M.I.M. was responsible for the submission; J.M.S., B.W., and S.S. were involved in the diagnosis and management of the patient and provided critical input to the manuscript throughout. F.S. was involved in the preparation of histology images. All authors reviewed and approved the manuscript's final version and agreed to accountability of all published content.

## Data Availability

Data sharing is not applicable to this article as no datasets were generated or analyzed during the current study.
